# Keratinocyte Progenitor Cells Reside in Human Subcutaneous Adipose Tissue

**DOI:** 10.1371/journal.pone.0118402

**Published:** 2015-02-25

**Authors:** Toshio Hasegawa, Atsushi Sakamoto, Akino Wada, Tatsuo Fukai, Hideo Iida, Shigaku Ikeda

**Affiliations:** 1 Department of Dermatology and Allergology, Juntendo University Graduate School of Medicine, 2-1-1 Hongo, Bunkyo-ku, Tokyo 113-8421, Japan; 2 Atopy (Allergy) Research Center, Juntendo University Graduate School of Medicine, 2-1-1 Hongo, Bunkyo-ku, Tokyo 113-8421, Japan; Center for Molecular Biotechnology, ITALY

## Abstract

The differentiation of adipose-derived stem cells (ASCs) towards epithelial lineages has yet to be demonstrated using a standardized method. This study investigated whether keratinocyte progenitor cells are present in the ASC population. ASCs isolated from subcutaneous adipose tissue were cultured and examined for the expression of the keratinocyte progenitor markers p63 and desmoglein 3 (DSG3) by immunofluorescence microscopy and flow cytometry. In addition, p63 and DSG3 expression levels were assessed before and after differentiation of ASCs into adipocytes by real-time PCR and western blot analysis, as well as in subcutaneous adipose tissue by real-time reverse transcriptase polymerase chain reaction. Both markers were expressed in ASCs, but were downregulated after the differentiation of ASCs into adipocytes; p63-positive cells were also detected in subcutaneous adipose tissue. ASCs co-cultured with human fibroblasts and incubated with all-trans retinoic acid and bone morphologic protein 4 showed an upregulation in DSG3 level, which was also increased in the presence of type IV collagen. They also showed an upregulation in cytokeratin-5 level only in the presence of type IV collagen. These results provide the demonstration that keratinocyte progenitor cells reside in subcutaneous adipose tissue.

## Introduction

The skin is a multilayered organ that protects the organism against external environmental stressors. Stem cell populations within the skin including the interfollicular epidermis, hair follicles, sebaceous glands, and sweat glands not only maintain skin homeostasis but also repair damaged areas. Stem cells in the basal layer of the epidermis give rise to short-lived progenitors, which amplify the keratinocyte population and migrate upwards as they differentiate [1]. In contrast, hair follicle stem cells can behave as multipotent stem cells during physical injury and participate in re-epithelialization [2–4]. Most notably, bulge stem cells contribute to the repair of the interfollicular epidermis when it has sustained damage [3, 5–7].

Stem cells include embryonic stem cells, induced pluripotent stem cells and adult stem cells. There are no ethical concerns related to the use of the latter. Among them, multipotent mesenchymal stem cells (MSCs) are non-hematopoietic cells of mesodermal origin that are present postnatally in various organs and connective tissues. Adipose-derived stem cells (ASCs) are among the most promising MSC populations for therapeutic applications, since human adipose tissue is easily obtained in large quantities with little donor site trauma [8]. Although ASCs can potentially generate an infinite number of somatic cells of any type, their differentiation towards epithelial lineages has yet to be demonstrated by a standardized method. The identification of keratinocyte progenitor cells is necessary and their potential for differentiation from ASCs must be determined before they can be considered for use in skin regeneration. The aim of this study was to investigate whether keratinocyte progenitor cells are present in ASC populations.

## Materials and Methods

### Ethics statement

Informed, written consent was obtained from all study participants. The documents of participant consent are preserved in each patient’s medical records. The study protocol was approved by the Ethics Committee of Juntendo University. Data were analyzed in blinded fashion and procedures were carried out according to the principles of the Declaration of Helsinki.

### Isolation and culture of ASCs

After obtaining the consent, subcutaneous adipose tissue was obtained from disease-free donors under local anesthesia and was washed extensively with phosphate-buffered saline (PBS). The extracellular matrix was digested at 37°C for 45 min with collagenase (SERVA Electrophoresis, Heidelberg, Germany). Enzyme activity was neutralized by adding control medium (Dulbecco’s modified Eagle’s Medium (DMEM) (Gibco, Life Technologies, Carlsbad, CA, USA) containing 10% fetal bovine serum (FBS)); the cell suspension was filtered through a 40-μm nylon mesh to remove debris and centrifuged at 1200 rpm for 10 min to obtain a high-density stromal vascular fraction pellet, which was resuspended in 5 ml control medium. The resulting cell population consisting mostly of ASCs were seeded in bare 60-cm^2^ culture dishes and maintained at 37°C in a humidified atmosphere of 5% CO_2_ in control medium.

After 7 days of culture, cells were harvested using a standard trypsinization protocol (0.25% trypsin/ethylenediaminetetraacetic acid [EDTA] for 3 min), washed with PBS, and incubated with FITC-conjugated anti-human antibodies against cluster of differentiation (CD)34, CD44, CD90, and CD105 (all from Becton-Dickinson, San Diego, CA, USA), for 30 min. Labeled cells were washed and analyzed using a FACSCalibur flow cytometer (BD Biosciences, San Jose, CA, USA).

### Adipogenic differentiation of ASCs in vitro

ASCs were tested for their ability to differentiate into adipocytes. Cells were plated in 6-well plates at a density of 3 x 10^5^ cells/well and allowed to adhere for more than 24 h in stromal medium (DMEM/Ham’s F12 containing 10% FBS and antibiotics) at 37°C in a CO2 incubator. When cells reached between 80% and 90% confluence, the medium was replaced first with differentiation medium (DMEM/Ham’s F12 containing 3% FBS, 3-isobutyl-1-methylxanthine, biotin, panthothenate, rosiglitazone, dexamethasone, and human insulin) [9] for 3 days, and then with adipogenic medium, which had the same composition as the differentiation medium but without 3-isobutyl-1-methylxanthine [9]. The adipogenic medium was changed every 3 days until mature adipocytes were obtained (day 12).

Adipogenic differentiation was assessed by Oil Red O staining. The degree of staining was quantified by destaining the cells with 100% isopropanol for 15 min and mesuring the optical density of the solution at 540 nm. Differentiation was also confirmed by real-time PCR detection of *adiponectin*, *leptin*, *peroxisome proliferator-activated receptor γ2* (*PPARγ2*), *lipoprotein lipase* (*LPL*), and *fatty acid-binding protein 4* transcript expression. Total RNA was extracted from cultured cells using the RNeasy Plus Micro kit (Qiagen, Hilden, Germany) according to the manufacturer’s protocol. A total of 2 μg RNA was converted to cDNA using the ReverTra Ace qPCR RT kit (Toyobo, Osaka, Japan). TaqMan Master Mix (Applied Biosystems, Foster City, CA, USA) was used to amplify 1 μg cDNA for 45 cycles on a Step One Plus system (Applied Biosystems). The expression of *adiponectin*, *leptin*, *PPARγ2*, *LPL*, and *fatty acid-binding protein 4* (using primers Hs00605917_m1, Hs00174497_m1, Hs01115513_m1, Hs00173425_m1, and Hs00173425_m1, respectively; Applied Biosystems) was normalized to *β-actin* levels, and the comparative cycle threshold (Ct) method using the formula 2^-ΔΔCt^ was used to calculate relative mRNA levels.

### Immunocytochemical detection of keratinocyte progenitor cell markers

The expression of keratinocyte markers in ASCs was detected by immunocytochemistry. Undifferentiated ASCs were fixed for 30 min in 4% paraformaldehyde at room temperature, and blocked with PBS containing 0.1% Triton X-100 and 10% goat serum (Jackson ImmunoResearch Laboratories, West Grove, PA, USA) for 1 h at room temperature before overnight incubation at 4°C with primary antibodies against p63 and desmoglein 3 (DSG3) (both at 1:1000, from Santa Cruz Biotechnology, Santa Cruz, CA, USA). Samples were washed, then incubated with secondary antibody (1:1000; Life Technologies) for 45 min at room temperature in the dark, then washed three times in PBS. Nuclei were counterstained with 4’,6-diamidino-2-phenylindole (Vector Laboratories, Burlingame, CA, USA). Samples were visualized under a Keyence BZ-9000 fluorescence microscope (Osaka, Japan). Normal human epidermal keratinocytes (NHEKs) were used as a positive control.

### Keratinocyte progenitor cell marker detection by flow cytometry

Undifferentiated ASCs were harvested using a standard trypsinization protocol, washed with PBS, and fixed with 4% formaldehyde in PBS for 10 min at 37°C. Ice-cold methanol was added and the cell suspension was mixed and left at -20°C for 30 min. The cells were then centrifuged, washed with 1% bovine serum albumin (Sigma-Aldrich, St. Louis, MO, USA) in PBS for 10 min, and incubated with phycoerythrin-conjugated antibody against p63 (1:800; Cell Signaling Technology, Danvers, MA, USA) and an FITC-conjugated antibody against DSG3 (1:1000; Abcam, Cambridge, MA, USA) for 30 min. Cells were washed and analyzed by flow cytometry with NHEKs used as positive control.

### Real-time PCR analysis of *p63* and *DSG3* mRNA expression in ASCs

The expression of keratinocyte lineage markers before and after the differentiation of ASCs into adipocytes was evaluated by real-time PCR. Total RNA was extracted from cultured cells on days 1 and 12 using the RNeasy Plus Micro kit according to the manufacturer’s protocol. A total of 2 μg RNA was converted to cDNA using the ReverTra Ace qPCR RT kit (Toyobo, Osaka, Japan). TaqMan Master Mix (Applied Biosystems, Foster City, CA, USA) was used to amplify 1 μg cDNA for 45 cycles on a Step One Plus system (Applied Biosystems). The expression of *p63* and *DSG3* (using primers Hs00978343_m1 and Hs00951897_m1, respectively; Applied Biosystems) was normalized to *β-actin* levels, and the comparative cycle threshold (Ct) method using the formula 2^-ΔΔCt^ was used to calculate relative mRNA levels.

### Quantitative analysis of p63 and DSG3 expression in ASCs by western blotting

The expression of keratinocyte markers before and after differentiation of ASCs into adipocytes was assessed by western blotting. ASCs were lysed in IGEPAL Nonidet P-40 in the presence of Halt Protease and Phosphatase Inhibitor Cocktail (both from Sigma-Aldrich). Proteins were separated by 10% Tris-glycine SDS-PAGE (Bio-Rad, Hercules, CA, USA) under denaturing conditions and transferred to a nitrocellulose membrane. After blocking with 3% bovine serum albumin in Tris-buffered saline, the membrane was incubated with primary antibodies against p63 (Cell Signaling Technology) and DSG3 (Santa Cruz Biotechnology) overnight at 4°C. The blot was proved for β-actin using a monoclonal antibody (1:2000; BioLegend, San Diego, CA, USA) as a loading control. The membrane was then washed, incubated with anti-mouse or -rabbit peroxidase-conjugated secondary antibody (1:1000; Santa Cruz Biotechnology and Cell Signaling Technology, respectively) at room temperature for 45 min, and developed with Luminate Forte western horseradish peroxidase substrate (Merck Millipore, Billerica, MA, USA).

### Detection of keratinocyte progenitor cells in subcutaneous adipose tissue

Subcutaneous adipose tissue was collected from donors under local anesthesia. Total RNA was purified from the tissue, with normal human keratinocytes serving as positive controls, using the RNeasy Plus Micro kit. Reverse transcriptase (RT)-PCR was performed with cDNA synthesized using the Moloney murine leukemia virus (M-MLV) RT kit (Invitrogen). The following components were included in the reaction: 1μl oligo (dT)_12–18_ (500 μg/ml), 1 μl of 10 mM dNTP mix (both from Invitrogen), 50 ng total RNA, and sterile distilled water to 12 μl. The mixture was incubated at 65°C for 5 min and immediately chilled on ice, and 4 μl of 5x first-strand systhesis buffer, 2 μl of 0.1 M dithiothreitol, and 1 μl RNaseOUT recombinant ribounclease inhibitor (40 U/μl) (Invitrogen) were added. The mixture was incubated at 37°C for 2 min and immediately chilled on ice; 1 μl of 200 U M-MLV RT was added and the mixture was incubated for 10 min at 25°C, followed by 50 min at 37°C and 15 min at 70°C. To remove RNA complementary to the cDNA, 1 μl of 2 U *Escherichia coli* ribonuclease H (Invitrogen) was added, followed by incubation at 37°C for 20 min. The amplification reaction consisted of 45 μl Platinum PCR SuperMix High Fidelity (Invitrogen), 200 nM primer solution, 100 ng genomic DNA template, and 1 U Platinum Taq DNA polymerase (Invitrogen). The following primers were synthesized by Invitrogen: for p63, 5’-GGT CCC CAC AGA GCA AGA-3’ and 5’-TGC AAT GAC AGC CCT TGA-3’; for DSG3, 5’-CAA AGC TGC CTC AAA TGT CA-3’ and 5’-TGC AAA CTG CAT CTT TTT CG-3’; and for the positive control glyceraldehyde-3-phosphate dehydrogenase, 5’-TGG GCT ACA CTG AGC ACC AG-3’ and 5’-CAG CGT CAA AGG TGG AGG AG-3’. The reaction conditions were as follows: 94°C for 2 min; 35 cycles of 94°C for 30 s; 58°C, 61°C, and 64°C for 30 s each; 68°C for 30 s; and 72°C for 5 min. PCR products were resolved by electrophoresis on 2% and 1.2% agarose gels (Nippon Gene, Tokyo, Japan) in 1x TAE buffer (40 mM Tris; 20 mM acetic acid; and 1 mM EDTA, pH 8.0). For each pair of gene-specific primers, semi-logarithmic plots of the intensity of amplified DNA fragments as a function of cycle number were used to determine the range of cycles over which linear amplification occurred, and the number of PCR cycles was kept within this range.

### Differentiation of ASCs into keratinocyte-like cells

A co-culture system was used to differentiate ASCs into keratinocyte-like cells. Normal human dermal fibroblasts (5 x 10^4^ cells) were seeded in the bottom chamber of 6-well plates and cultured in DMEM containing 10% FBS for 24 h, while ASCs (10^5^ cells) were seeded on 0.4-μm Millicell hanging cell culture inserts (Merck Millipore) coated with type IV collagen (Nitta Gelatin, Osaka, Japan) that were placed in the plates; 1 μM all-trans retinoic acid (ATRA) (Sigma-Aldrich) was added to the upper chamber, and cells were cultured for 72 h. Bone morphogenetic protein 4 (BMP4) (R&D Systems, Minneapolis, MN, USA) was added at 25 ng/ml to the upper chamber. After 4 days, the ATRA- and BMP4-containing medium was replaced with keratinocyte serum-free medium (Invitrogen) for 7 more days. ASC remnants were removed and analyzed by real-time PCR for *DSG3* and *cytokeratin 5* (*K-5*) expression. ASCs cultured without ATRA or BMP4 or with neither, and ASCs co-cultured with fibroblasts on non-type IV collagen-coated transwell inserts were used as controls.

### Cell viavility assay

Cell viability of ASCs co-cultured with fibroblasts on type IV collagen coating was assessed by collecting the media from the 6-well plates and adding 270 μl 3-(4,5-di-methylthiazol-2-yl)-2,5-diphenyltetrazolium bromide (MTT) to each well and incubating the plates for 1 h at 37°C. The supernatant was removed and 1 ml of dissolving solution (Cayman Chemical, Ann Arbor, Michigan, USA) was added to each well. Absorbance was read at 570 nm using a microplate reader.

### Statistical analysis

Data were analyzed by analysis of variance and are presented as mean ± SD. Differences were considered statistically significant at P ≤ 0.05.

## Results

### Isolation and adipogenic differentiation of ASCs

**Fig 1:**
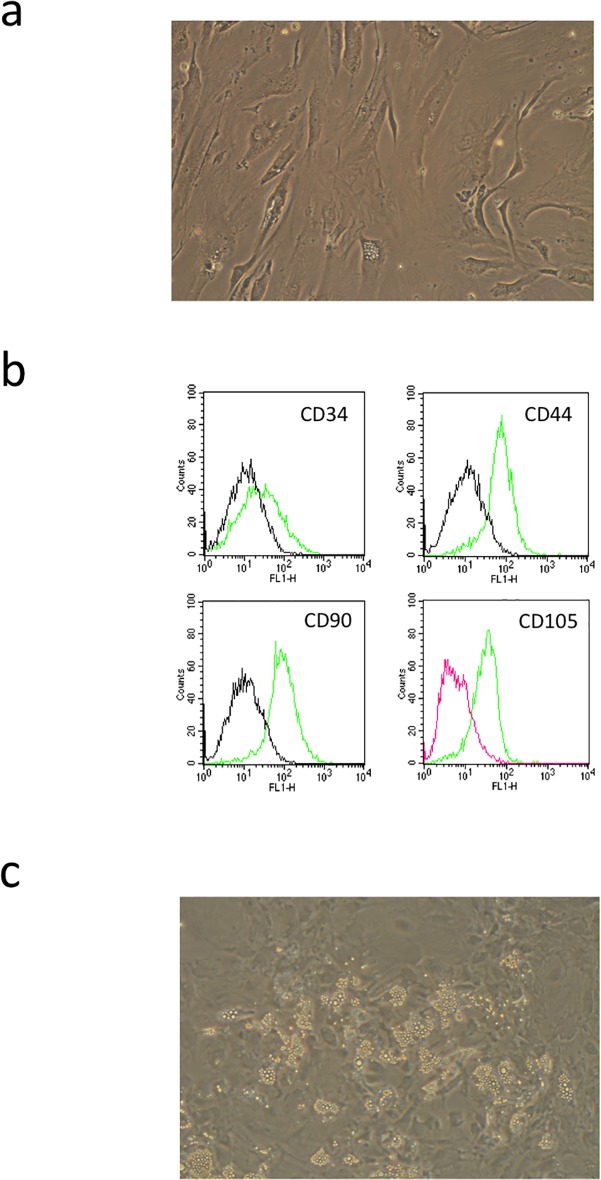
Isolation and adipogenic differentiation of ASCs. (a) ASCs isolated from human subcutaneous adipose tissue adhered to the dish and were spindle- or stellate-shaped. (b) ASCs were positive for the markers CD34, CD44, CD90, and CD105 by flow cytometry. (c) ASCs cultured in adipogenic medium exhibited a greater variety of cell morphologies and exhibited a time-dependent increase in intracellular lipid vacuoles, as seen by Oil Red O staining.

ASCs adhered to the dish and became spindle- or stellate-shaped cells (Fig. 1A) that were positive for CD34, CD44, CD90, and CD105, as determined by flow cytometry (Fig. 1B).

ASCs cultured in adipogenic medium exhibited a greater variety of cell morphologies and a time-dependent increase in intracellular lipid vacuoles that appeared after only 4 days, with cell size and the number of lipid vacuoles increasing steadily thereafter (Fig. 1C). The absorbance at 540 nm of Oil Red O-stained cells increased markedly over this time course, while *adiponectin*, *leptin*, *PPARγ2*, *LPL*, and *fatty acid-binding protein 4* transcript levels were upregulated. These results demonstrate the differentiation of ASCs into adipocytes (data not shown).

### ASCs express keratinocyte markers

**Fig 2:**
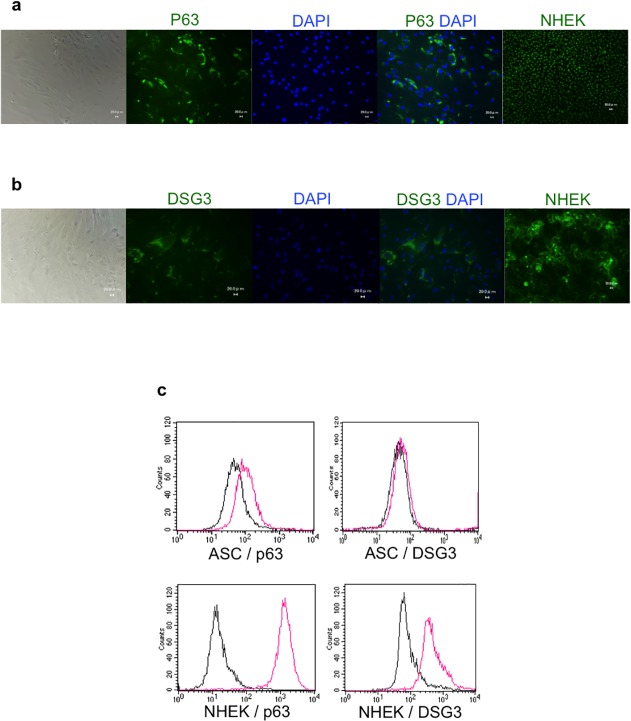
Keratinocyte marker expression in ASCs. ASCs cultured in chambered slides were fixed and labeled with antibodies against p63 and DSG3. Undifferentiated ASCs and NHEKs (positive control) expressed (a) p63 and (b) DSG3 (both visible by green fluorescence). Scale bars, 20 μm. (c) The expression of p63 and DSG3 in ASCs and NHEKs was detected by flow cytometry.

After 7 days of culture, ASCs expressed p63 and DSG3, as determined by immunofluorescence microscopy (Fig. 2A, B) and flow cytometry (Fig. 1C); control NHEKs were also positive for these markers. Without intracellular staining procedure, p63 and DSG3 were not detected by flow cytometry (data not shown). These observations were consistent across three independent experiments performed using ASCs from three different donors.

### Keratinocyte marker expression decreases upon differentiation of ASCs into adipocytes

**Fig 3:**
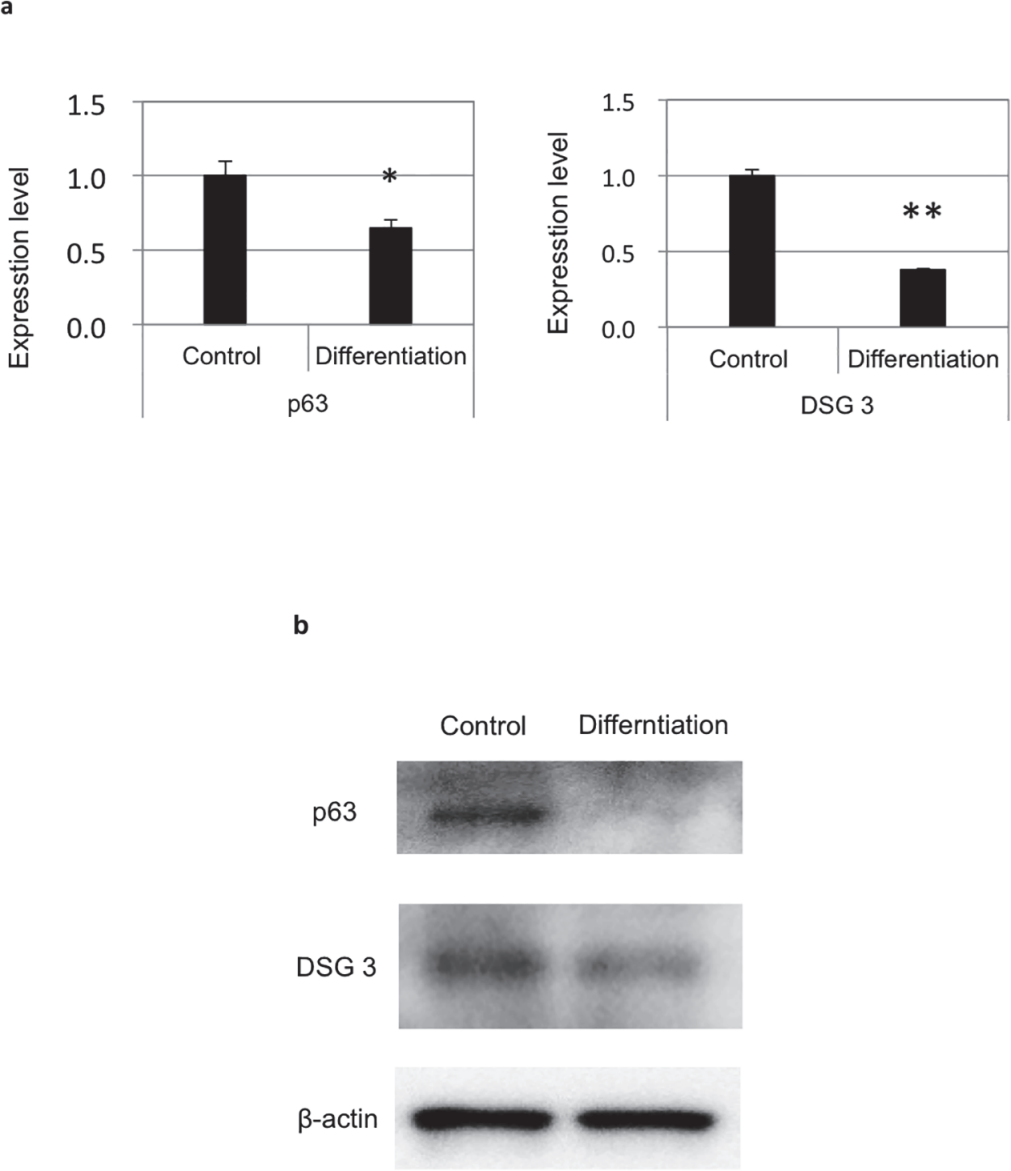
Downregulation of keratinocyte marker expression in ASCs after their differentiation into adipocytes. The expression of p63 and DSG3 was measured by (a) quantitative real-time PCR (relative to glyceraldehyde-3-phosphate dehydrogenase) and (b) by western blotting (relative to β-actin) in undifferentiated ASCs and those that had differentiated into adipocytes. Both markers were expressed at lower levels after differentiation.

Real-time PCR (Fig. 3A) and western blot (Fig. 3B) analyses revealed that undifferentiated ASCs were positive for p63 and DSG3. In addition, the expression of these markers was reduced after differentiation of ASCs into adipocytes as compared to undifferentiated cells. These results were consistent across three experiments performed using ASCs from three different donors.

### Keratinocyte marker expression in human subcutaneous adipose tissue

**Fig 4:**
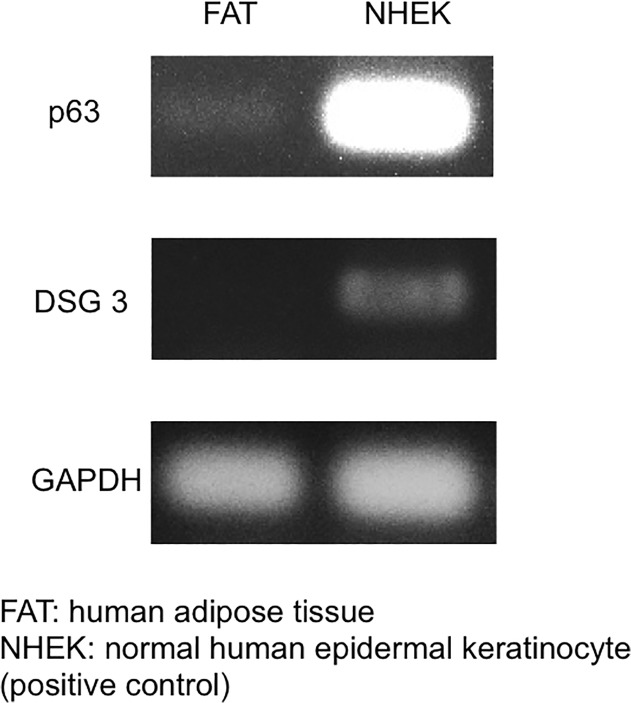
Keratinocyte marker expression in human subcutaneous adipose tissue. The *p63* transcript (but not that of *DSG3*) was detected in human subcutaneous adipose tissue by RT-PCR but at a lower level than in NHEKs.

Human subcutaneous adipose tissue expressed *p63* but a lower level than in NHEKs, as determined by RT-PCR (Fig. 4); however, *DSG3* mRNA was not detected. These results demonstrate that p63-positive keratinocyte progenitor cells are present in adipose tissue.

### ASCs transdifferentiate into keratinocyte-like cells

**Fig 5:**
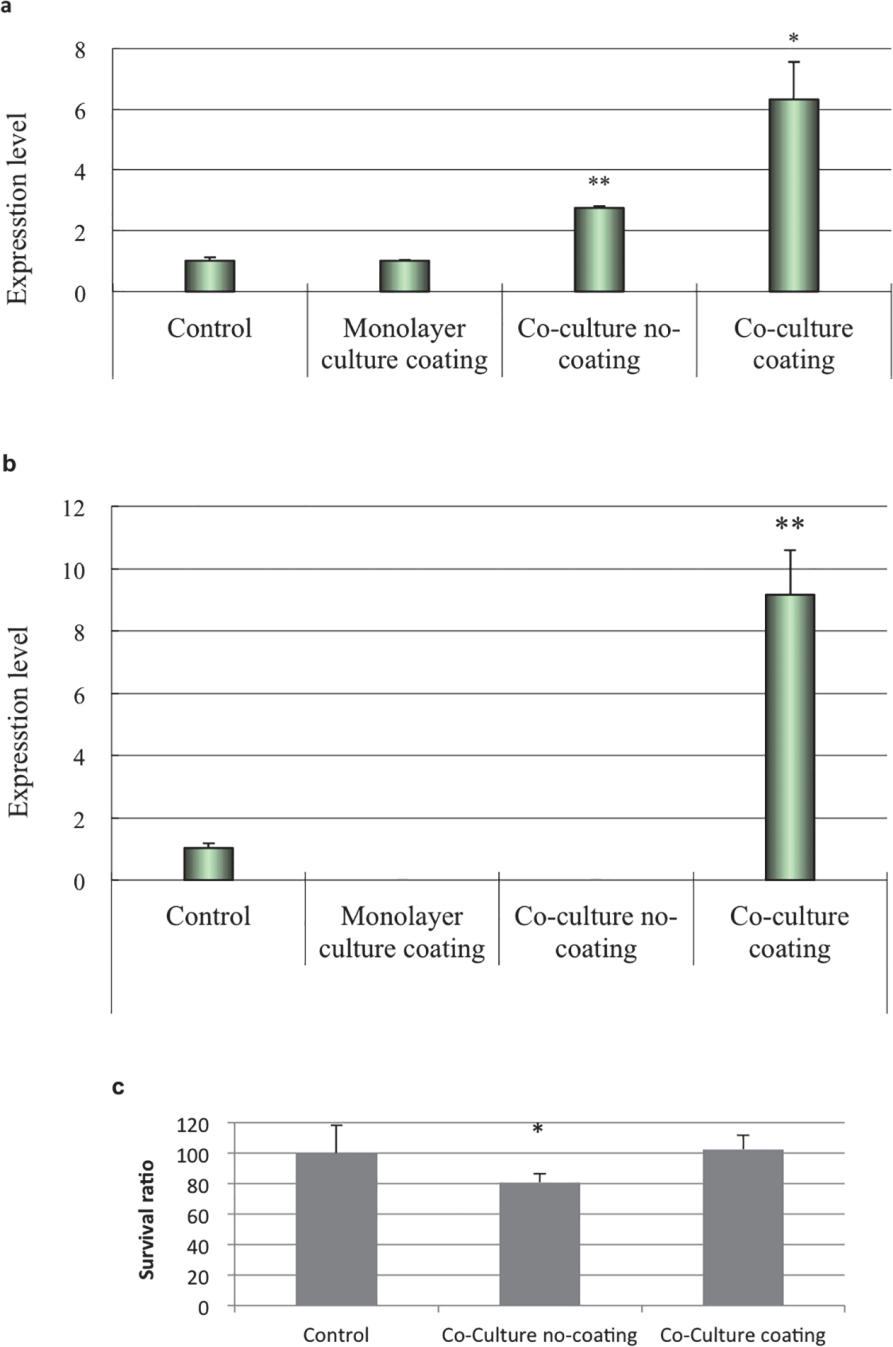
Transdifferentiation of ASCs into keratinocyte-like cells. The transdifferentiation of ASCs into keratinocyte-like cells was assessed by detecting (a) *DSG3* and (b) *K-5* mRNA expression by real-time PCR. Treatment with ATRA and BMP4 had no effect on *DSG3* or *K-5* expression in ASCs cultured as a monolayer, but induced an increase in the transcript expression of *DSG3* gene in ASCs co-cultured with fibroblasts on non-type IV collagen-coated transwell inserts. Type IV collagen coating increased *DSG3* and *K-5* expression in ASCs co-cultured with fibroblasts. (b) Cell viability in co-cultures with or without type IV collagen coating was assessed by the MTT assay. The type IV collagen coating had no adverse effects on cell viability.

To assess the epithelial differentiation potential of ASCs co-cultured with fibroblasts, the expression of *DSG3* and *K-5* was evaluated by real-time PCR. Treatment with ATRA and BMP4 had no effect on the expression of *DSG3* and *K-5* in ASCs cultured separately as monolayers (Fig. 5A, B). ASCs co-cultured with fibroblasts on non-type IV collagen-coated transwell inserts had high level of DSG3 expression; type IV collagen coating, moreover, increased the expression of *DSG3* and *K-5*. These results suggest that the extracellular matrix and dynamic cross-communication between fibroblasts and ASCs modulate ASC transdifferentiation.

Cell viability was assessed by the MTT assay. ASCs were viable in the co-culture system and also when grown on type IV collagen coating (Fig. 5C), indicating that both conditions induce morphological changes in ASCs without adversely affecting cell viability.

## Discussion

Human bone marrow-derived MSCs can differentiate into functional epithelial-like cells in vitro [10, 11]. However, MSCs can also be isolated from adipose tissue. The advantages of using adipose tissue-derived ASCs, which are one type of MSC, include their abundance in a given donor and the ease with which they can be obtained using relatively noninvasive methods. On the other hand, the differentiation of ASCs into unexpected lineages is a significant concern. The present study investigated whether ASCs have the potential to differentiate into keratinocytes.

ASCs were isolated from human subcutaneous adipose tissue and were cultured for 2 weeks to assess their potential for differentiating into adipocytes, which was evaluated by the detection of epidermal marker expression. The p63 protein has been proposed as an important transcription factor in the development of squamous epithelia, and there is substantial evidence for p63 as a stem cell determinant in epithelial cell types [12, 13]. In human epidermis and epidermal cultures, p63 expression is restricted to cells with high proliferative potential in the basal layer [14]. DSG3 is a cadherin-type cell membrane protein that mediates cell-cell adhesion by coupling to keratin intermediate filaments in the skin, which depends on keratinocyte location and maturation. Based on the present observations that p63 and DSG3 are expressed in ASCs and their expression is downregulated after the differentiation of ASCs into adipocytes, we speculate that ASCs are keratinocyte stem/progenitor cells. Interestingly, DSG3 as well as p63 were detected intracellularly in ASCs by flow cytometry.

It was recently reported that ASCs co-cultured with keratinocytes differentiated into keratinocyte-like cells [15]. In the present study, ASCs were co-cultured with human fibroblasts; under these conditions, ATRA and BMP4 treatment or the presence of type IV collagen stimulated increased expressions of *DSG3* and *K-5*. In contrast, type I collagen had no effect on keratinocyte progenitor cell marker expression (data not shown). Basement membrane proteins such as type IV collagen can accelerate the differentiation of various types of cells [16]. Thus, the presence of fibroblasts combined with the extracellular matrix stimulate the differentiation of ASCs into keratinocytes.

Wound healing is a complex process involving many well-coordinated events such as inflammation, cell proliferation and migration, matrix production, and angiogenesis. The balance between proliferation and differentiation–which controls tissue homeostasis in the epidermis–is shifted towards proliferation upon skin injury and must be restored after wound healing is completed. Wound closure is mediated not only by contractile granulation tissue produced by fibroblasts and macrophages, but also involves re-epithelialization via the proliferation and migration of keratinocytes at the wound margin [17]. Full-thickness wounds resulting from tumor resection or injury are often treated with skin grafts that may not survive due to persistent infection, inflammation, and bleeding during the early postoperative course. However, in some cases, epithelialization begins long after the disappearance of the grafted skin, which should contain not only a normal epidermis but also a sufficient number of keratinocyte stem cells for long-term epidermal renewal. However, in addition to the basal stem cells required for normal epithelial homeostasis, other progenitors may also contribute to epithelial layer regeneration. ASCs may be mobilized to sites of injury where a subset differentiate into keratinocytes. Additionally, ASCs can also enhance wound repair by creating a microenvironment that promotes local regeneration of cells in the affected tissue.

## Conclusion

This study demonstrated that keratinocyte progenitor cells reside in human subcutaneous adipose tissue, suggesting that this tissue has the capacity to produce keratinocytes. The results also revealed that both co-culturing and an extracellular matrix are needed for ASC differentiation.
